# The validity and reliability of Persian version of smartphone addiction questionnaire in Iran

**DOI:** 10.1186/s13011-021-00407-5

**Published:** 2021-09-17

**Authors:** Zahra Shaahmadi, Touraj Ahmadi Jouybari, Bahare Lotfi, Abbas Aghaei, Reza Ghanei Gheshlagh

**Affiliations:** 1 Clinical Research Development Center, Imam Khomeini and Mohammad Kermanshahi and Farabi Hospitals, Kermanshah University of Medical Sciences, Kermanshah, Iran; 2 Social Determinants of Health Research Center, Research Institute for Health Development, Kurdistan University of Medical Sciences, Sanandaj, Iran

**Keywords:** Validity, Reliability, SAS questionnaire, Smartphone addiction, Confirmatory factor analysis

## Abstract

**Background:**

Smartphone addiction is one of the most important forms of technology addiction that has attracted the attention of all countries around the world. Many studies have been conducted in Iran on cellphone addiction among different groups. There is a necessity to have a native scale for measuring smartphone addiction in particular. Therefore, this study aimed to localize the smartphone addiction questionnaire in Iran (in the Persian language).

**Methods:**

To assess the validity and reliability of the Persian version of the smartphone addiction scale (SAS), the questionnaire was first provided based on the standard back-translation method. Next, content validity ratio (CVR), content validity index (CVI), and face validity was evaluated for translated questionnaire. After making the necessary changes, the questionnaire was given to the community samples and was then reviewed using confirmatory factor analysis of questions grouping. Finally, the reliability of the questionnaire was investigated by the test-retest method.

**Results:**

The CVR and CVI values of all questions were within the acceptable range. Only some of the questions in the original SAS version titled Twitter and Facebook were changed to Instagram and telegram according to experts. Internal consistency and concurrent validity of the questionnaire were confirmed by Cronbach’s alpha of 0.951. The mean correlation coefficient between the responses of the subjects, who received the questionnaire twice, was 0.946 (0.938–0.954). The grouping of questions in the subscales was changed from the original SAS version because the fitting indexes, obtained from the confirmatory factor analysis test (for example CMIN/DF greater than 5 units and RMSEA of approximately 0.07), were not acceptable.

**Conclusion:**

The results showed that the Iranian version of the cellphone addiction questionnaire can be used as a valid, with minimal modification, tool for determining the level of smartphone addiction among Persian speakers.

## Background

Technology and the growth of industry certainly affect human health. Today, there is a significant increase in the excessive use of cellphones [1]. Smartphones are not only cellphones anymore, but also a means of accessing many of the latest technologies including the Internet at any time and place. Therefore, with this capability, many people seek to develop and use it [2, 3]. In such a situation, smartphone addiction can be considered as the most important form of technology addiction that has attracted the attention of countries around the world [4–7]. Studies by the Korea National Intelligence Association in 2012 showed that smartphone addiction and internet addiction were 8.4 and 7.7%, respectively [4].

In Korea, simple statistical methods were used to evaluate smartphone addiction. However due to the ambiguity and limitations of this method, Kwon et al. designed a smartphone addiction scale (SAS) for the first time and evaluated its reliability and validity in Kangwon, Korea [8]. This questionnaire was developed according to the many aspects of smartphone addiction and the K-scale (Korean self-diagnostic program for Internet addiction), derived from the Kimberly Young Internet Addiction Scale. Then, it has been revised and finalized using factor analysis [9–12]. As a result of this research, Cronbach’s alpha showed 96.6% internal consistency of the items of this 33-item questionnaire [8]. The reliability and validity of this questionnaire in Turkey (2014) and Malaysia (2015) have also been evaluated by Akin Ahmed et al. and Ching et al., respectively [13, 14]. There have been many studies of cellphone addiction in Iran across different groups [15–20], but there is no exact study due to the lack of a native scale for measuring smartphone addiction in particular. Therefore, the present study aimed to localize the SAS questionnaire among the Iranian population (Kermanshah city).

## Methods

### Study design

This cross-sectional methodological study was conducted to evaluate the psychometric properties of the Persian version of the Smartphone Addiction Scale.

### Participants

The study samples included 360 staff and clients of two general and large hospitals of Kermanshah (Imam Khomeini Hospital and Imam Reza Hospital) who were selected by convenience sampling. Since these hospitals are referral centers in western Iran, their clients (Patients and accompanying person) cover almost every spectrum of society, so it was attempted to select generalizable samples from the hospital staff and clients.

### Translation

After obtaining permission from the original designer, the English version of the Smartphone Addiction Scale was translated into Persian by two independent translators. The two translated versions were compared and a single version was compiled by the research team. The original version of the questionnaire was prepared by comparing and combining the two versions according to the agreement of the authors. Then, the items that needed to be corrected were reviewed and corrected by both translators through comparing two translated texts and providing feedback to two translators. The final translated text was given to another translator, who was fluent in English, and finally, the translated text was matched to the original English text and provided to all three translators. Finally, minor cases were corrected in coordination with all translators.

### Face validity

After evaluating the content validity of the questionnaire and making the necessary corrections, the face validity of the questionnaire is accessed by 5 experts in the study subject, and 22 target groups, who were selected by easy sampling from hospital staff. They were asked to comment on the difficulty or simplicity of the questions, their meaning, or any ambiguity in the questions, phrases or words after reading and completing the questionnaire. The corrections are made in case of necessity as the necessary questions are received.

### Content validity

An expert panel consisting of 15 experts in the field of study is used to assess the content validity of the questionnaires: 5 in health education and health promotion, 4 in epidemiology, 4 in psychology, and 2 in sociology. To determine the content validity ratio (CVR), the experts were asked to examine each question on a three-part range of “essential”, “useful but unnecessary” and “unnecessary”. Moreover, the content validity index (CVI) was separately evaluated by experts through three criteria of simplicity, appropriateness and certainty based on a four-part spectrum (for example, in terms of simplicity, quite simple, somewhat complex and complex) for each question and the corresponding ratings were given. Finally, the content validity indices were measured and judged by each question, each subscale and the whole questionnaire.

### Data analysis

Exploratory factor analysis was used to evaluate the construct validity. Adequacy of sampling was assessed by Kaiser-Meyer-Olkin (KMO) and Bartlett test. Extraction of latent agents was performed using the Maximum Likelihood method and Varimax rotation by SPSS software version 25. The cut-off point of factor loading was considered to be 0.30. 360 new available samples were selected for confirmatory factor analysis (CFA). At this stage, fit indicators such as the goodness of fit index (GFI), chi-square test (χ2), degrees of freedom (df), root mean square error of approximation (RMSEA), confirmatory fit index (CFI), standardized root mean square residuals (SRMR), and Tucker-Lewis index (TLI) fit indices were evaluated. Internal consistency with Cronbach’s alpha coefficient and instrument stability was calculated by the interclass correlation (ICC) with a Two-way mixed-effects model and absolute agreement with a 95% confidence interval.

## Results

### Participants’ characteristics

The samples included 175 females and 185 males with a mean age of 32.19 32 8.53 years. Of these, 74 were hospital staff, 56 were patients, 188 were patient’s accompanies and 42 were students. In terms of education, 29 people had primary and secondary education, 96 people had high school education and the rest had university education.

### Validity

The content validity of the questionnaire is evaluated by 11 experts from the panel (consist of 2 PhDs in Epidemiology, 3 PhDs in Health Education, 2 PhDs in Sociology, 1 MSc in Health Education, 1 Ph.D. in Health Policy, 1 Ph.D. in Health Care Management, 1 MSc in psychology). To determine the content validity ratio (CVR), the experts were asked to examine each question on a three-part range of “essential”, “useful but unnecessary” and “unnecessary”. Moreover, the content validity index (CVI) was separately evaluated by experts through three criteria of simplicity, appropriateness and certainty based on a four-part spectrum (for example, in terms of simplicity, quite simple, somewhat complex and complex) for each question and the corresponding ratings were given. Finally, the content validity indices were measured and judged by each question, each subscale and the whole questionnaire. The CVR values of all questions varied between 0.6–1 concerning the 11 experts in this study and it was above the established recommended levels (minimum validity was 0.59) [21], then all questions were considered essential and none were eliminated. The CVI value for all questions varied between 0.8–1, which is in the acceptable range. The scores of simplicity, appropriateness and certainty for all questions were 0.94, 0.91, and 0.98, respectively. However, some questions were changed according to the opinion of expert panel members. For example, Telegram and Instagram are used in this study instead of the original version for social networks such as Twitter and Facebook (Table 1).

**Table 1:** Results of the content validity indexes for the SAS questionnaire

Subscale	Question	CVR	Simplicity CVI	Appropriateness CVI	certainty CVI
**Daily life disturbance (q1-q5)**	Missing planned work due to smartphone use	1	1	1	1
	Having a hard time concentrating in class, while doing assignments, or while working due to smartphone use	0.8	0.8	0.8	0.8
	Experiencing lightheadedness or blurred vision due to excessive smartphone use	0.8	1	1	1
	Feeling pain in the wrists or at the back of the neck while using a smartphone	1	1	1	1
	Feeling tired and lacking adequate sleep due to excessive smartphone use	1	1	1	1
**Positive** a**nticipation (q6-q13)**	Feeling calm or cozy while using a smartphone	0.8	1	1	1
	Feeling pleasant or excited while using a smartphone	0.8	1	1	1
	Feeling confident while using a smartphone	0.8	1	0.6	1
	Being able to get rid of stress with a smartphone	0.8	1	0.8	1
	There is nothing more fun to do than using my smartphone.	0.8	1	1	1
	My life would be empty without my smartphone.	0.8	1	1	1
	Feeling most liberal while using a smartphone	0.6	0.8	1	1
	Using a smartphone is the most fun thing to do.	1	1	1	1
**Withdrawal (q14-q19)**	Won’t be able to stand not having a smartphone	0.8	1	0.8	1
	Feeling impatient and fretful when I am not holding my smartphone	0.8	1	1	1
	Having my smartphone in my mind even when I am not using it	0.8	0.8	0.8	1
	I will never give up using my smartphone even when my daily life is already greatly affected by it.	1	0.8	1	0.8
	Getting irritated when bothered while using my smartphone	0.6	1	0.8	1
	Bringing my smartphone to the toilet even when I am in a hurry to get there	0.8	0.8	0.8	1
**Cyberspace oriented relationship (q20-q26)**	Feeling great meeting more people via smartphone use	0.6	0.8	0.8	1
	Feeling that my relationships with my smartphone buddies are more intimate than my relationships with my real-life friends	0.6	0.8	0.8	1
	Not being able to use my smartphone would be as painful as losing a friend.	0.8	1	1	1
	Feeling that my smartphone buddies understand me better than my real-life friends	0.8	1	0.8	1
	Constantly checking my smartphone so as not to miss conversations between other people on Telegram or Instagram	1	1	1	1
	Checking SNS (Social Networking Service) sites like Telegram or Instagram right after waking up	1	1	1	1
	Preferring talking with my smartphone buddies to hanging out with my real-life friends or with the other members of my family	1	0.8	1	1
**Overuse (q27-q30)**	Preferring searching from my smartphone to asking other people	0.8	1	0.8	1
	My fully charged battery does not last for one whole day.	0.8	1	1	1
	Using my smartphone longer than I had intended	0.8	0.8	0.8	1
	Feeling the urge to use my smartphone again right after I stopped using it	1	1	0.8	1
**Tolerance (q31-q33)**	Having tried time and again to shorten my smartphone use time, but failing all the time	1	1	1	1
	Always thinking that I should shorten my smartphone use time	1	1	1	1
	The people around me tell me that I use my smartphone too much.	1	1	1	1

In exploratory factor analysis, the KMO criterion was 0.957 and the Bartlett sphericity test was significant (X2 = 15,748.6, p = < 0.001).

In the exploratory factor analysis, 6 factors were extracted that were able to explain 63.267% of the total variance (Table 2).

**Table 2:** Factors identified in exploratory factor analysis of the localized version of the SAS questionnaire

Factors	Eigenvalue	Rotation Sums of Squared Loadings		
		Total	% of Variance	Cumulative%
1	12.99	4.74	14.37	14.37
2	2.08	3.85	11.67	26.04
3	2.03	3.64	11.03	37.08
4	1.57	3.23	9.79	46.86
5	1.25	2.86	8.68	55.54
6	0.95	2,55	7.73	63.27

**Fig. 1:**
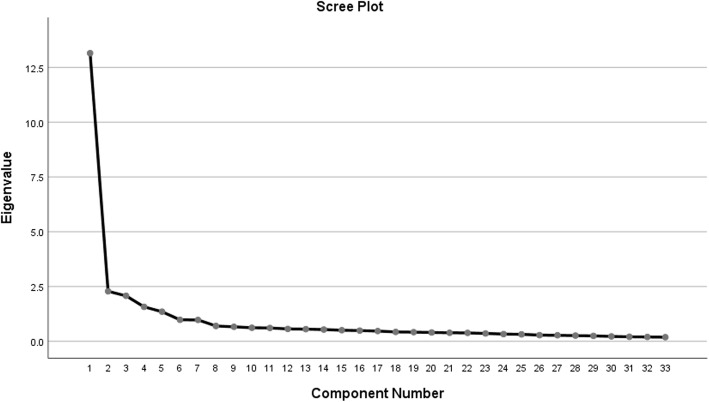
Screen plot of the exploratory factor analysis

Fig. 1 shows the screen plot of the exploratory factor analysis applied in this study. Also the grouping of questions in the Persian version of SAS questionnaire based on exploratory factor analysis is reported in Table 3.

**Table 3:** Grouping of question s in Persian version of SAS questionnaire based on exploratory factor analysis

Rotated Component Matrix^**a**^						
	Component					
	Daily life disturbance	Positive Anticipation	Withdrawal	Cyberspace oriented relationship	Overuse	Tolerance
**Q1**	0.625					
**Q2**	0.714					
**Q3**	0.797					
**Q4**	0.727					
**Q5**	0.718					
**Q6**		0.766				
**Q7**		0.747				
**Q8**		0.779				
**Q9**		0.729				
**Q10**			0.505			
**Q11**			0.649			
**Q12**			0.475			
**Q13**			0.638			
**Q14**			0.659			
**Q15**			0.746			
**Q16**			0.686			
**Q17**			0.629			
**Q18**			0.573			
**Q19**			0.306			
**Q20**				0.674		
**Q21**				0.713		
**Q22**				0.501		
**Q23**				0.733		
**Q24**				0.550		
**Q25**					0.660	
**Q26**					0.364	
**Q27**					0.773	
**Q28**					0.521	
**Q29**						0.511
**Q30**						0.540
**Q31**						0.683
**Q32**						0.777
**Q33**						0.613

Extraction Method: Principal Component Analysis

Rotation Method: Varimax with Kaiser Normalization

^a^ Rotation converged in 7 iterations

**Fig. 2:**
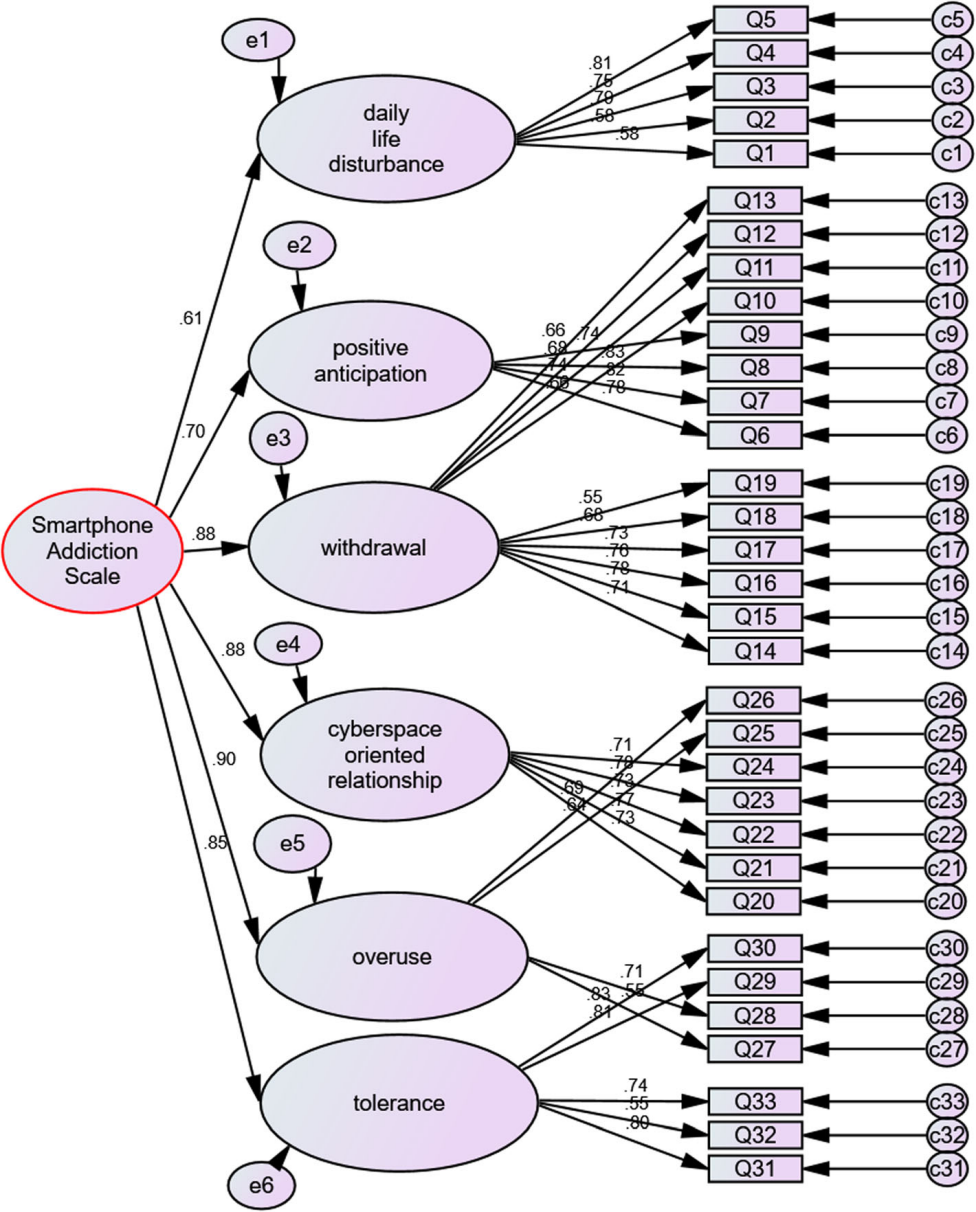
The final model. This model is the output of Amos software based on confirmatory factor analysis was performed on the results of the SAS Persian version. Arrows that fall out of their respective categories and are drawn to another category, the differences found between the original version of SAS and its Persian version are based on the confirmatory analysis factor

Confirmatory factor analysis was performed based on the new category, because many of the indexes of confirmatory factor analysis fitting based on initial classification (according to the original study of SAS) were not appropriate, for example, the Chi^2^ likelihood ratio statistic (CMIN/DF) was larger than 5 units and RMSEA was approximately equal to 0.07. In the next step, confirmatory factor analysis was performed based on the results of the Persian version of the factor analysis. Figure 2 depicts the output of Amos software based on exploratory factor analysis for this analysis for the Persian version of SAS questionnaire.

Comparison of the general indices of the results of the confirmatory factor analysis based on the results of the new classification of the localized SAS questionnaire with the general fitting indexes of the factor analysis model based on the initial classification showed that all indexes are improved. For example, the CMIN/DF and RMSEA indices were lower than 4 and 0.06, respectively (Fig. 2 and Table 4).

**Table 4:** General fitting indexes of the confirmatory factor analysis model based on the results of the new classification of Persian SAS questionnaire

RMSEA	PCFI	PNFI	CMIN/DF	TLI	CFI	RFI	IFI	P	DF	CMIN
0.058	0.837	0.812	3.882	0.901	0.909	0.872	0.910	< 0.001	489	1898.298

RMSEA: root mean square error of approximation, PCFI: parsimony comparative fit index, PNFI: Parsi-mony normed fit index, CMIN/DF: the Chi^2^ likelihood ratio statistic, TLI: Tucker-Lewis index, CFI: comparative fit index, RFI: relative fit index, IFI: incremental fit index, P: *p*-value, DF: degree of freedom, CMIN: Chi-square index

The Chi-square index (CMIN) has been decreased based on the results of the new classification of the localized SAS questionnaire, and other indices have been increased. The reliability calculated using Cronbach’s alpha coefficient was 0.951. The mean correlation coefficient between the responses of the subjects who received the questionnaire twice was 0.946 (0.938–0.954). Finally, the grouping of questions for the Persian version of SAS questionnaire is obtained based on this study as shown in Table 5.

**Table 5:** Final grouping of questions in subscales for Persian version of SAS questionnaire

Subscale	Questions based on Persian version of SAS	Questions based on original SAS
Daily life disturbance	Question 1–5	Question 1–5
Positive anticipation	Question 6–9	Question 6–13
Withdrawal	Question 10–19	Question 14–19
Cyberspace oriented relationship	Question 20–24	Question 20–26
Overuse	Question 25–28	Question 27–30
Tolerance	Question 29–33	Question 31–33

## Discussion

The purpose of this study was to localize the SAS questionnaire. The CVR and CVI values of all questions were within the acceptable range and none of the questions were eliminated. It should be mentioned that some of the questions in the original SAS version titled Twitter and Facebook were changed to Instagram and Telegram according to the authors. Internal consistency and concurrent validity of the questionnaire were confirmed with Cronbach’s alpha of 0.951. These values were reported for other previous studies as follows: Min Kwon et al. [8] (Original version of SAS questionnaire) obtained internal consistency and reliability of the questionnaire with Cronbach’s alpha of 0.967. Ching et al. [14] investigated the validity and reliability of the SAS questionnaire in Malaysia with Cronbach’s alpha of 0.94. The Cronbach’s alpha for validity and reliability of the shortened SAS version conducted by Bede C. Akpunne et al. [22] in Nigeria was 0.82. Khalily et al. [23] investigated the validity and reliability of the short version of SAS in Pakistan (Urdu language) with Cronbach’s alpha coefficient of 0.81 which was consistent with the original SAS version.

The value of Cronbach’s alpha in the present study is much closer to that of the original study. This can be explained by the fact that the target group in our study (employees, 20–60 years) was closer to the original study (in the original study, 18–53 years). The mean age of Ching et al.’s study [14] was 21 years for high school students. The age range of the participants in the study of Khalily et al. [23] was 18–20 years.

The grouping of questions in the subscales was changed from the original SAS version because the fitting indexes, obtained from the confirmatory factor analysis test, were not appropriate validating factors (e.g. CMIN/DF greater than 5 units and RMSEA of approximately 0.07). Therefore, exploratory factor analysis was applied and confirmatory factor analysis was utilized based on this analysis. The results showed that the values of the fitting indices were improved (CMIN/DF and RMSEA indices were less than 4 and 0.06, respectively) and changes were made to the question grouping, but none of the subscales and questions were deleted.

These changes were such that no changes were made to the “daily life disturbance” factor and constructs included questions number 1 to 5 in the original version. However, the changes of question numbers for other factors were as follows: “positive anticipation” included question numbers 6 to 9 while it was from question number 6 to 13 in the original version; “withdrawal” included question numbers 10 to 19 while it was from question number 14 to 19 in the original version; “cyberspace oriented relationship” included question numbers 20 to 24 while it was from question number 20 to 26 in the original version; “overuse” included question numbers 25 to 28 while it was from question number 27 to 30 in the original version, and “tolerance” included question numbers 29 to 33 while it was from question number 31 to 33 in the original version.

In the study conducted by Ching et al. [14], the validity and reliability of the SAS questionnaire are assessed by the Malaysian language and evaluated the questionnaire with correlation analysis, factor analysis and t-test. They concluded that the internal consistency and reliability of the questionnaire are confirmed by Alpha Cronbach of 0.94. They also showed that all constructs except positive anticipation constructs were related to the original version and the questionnaire was applicable by omitting the positive anticipation factor.

At first glance, the use of hospital population may be considered as a weakness in this study, but since the hospitals are two large and public hospitals in Kermanshah metropolis and the sampling among the hospital staff, patients and their companions as well as students, were taken, it can be said that the samples can be somewhat similar to the general population.

## Conclusion

Although there were slight changes in the Persian version of SAS questionnaire from the original questionnaire (which requires localization in a different culture), the results showed that the validity and reliability of the Persian version of SAS questionnaire were acceptable. These minor changes did not make much difference to the original SAS questionnaire questions.

## Data Availability

The datasets generated and/or analysed during the current study are not publicly available because the intellectual property is owned by the funding body. They may be available from the corresponding author on reasonable request subject to approval from the funding body.

## Abbreviations

CFA: confirmatory factor analysis
CFI: comparative fit index
CMIN: Chi-square index
CMIN/DF: the Chi^2^ likelihood ratio statistic
CVR: content validity ratio
CVI: content validity index
DF: degree of freedom
GFI: goodness-of-fit index
IFI: incremental fit index
NFI: normed Fit Index
PCFI: parsimony comparative fit index
PNFI: Parsi-mony normed fit index
RFI: relative fit index
RMSEA: root mean square error of approximation
SAS: smartphone addiction scale
SRMR: standardized root mean squared error
TLI: Tucker-Lewis index
